# What makes an article a must read in medical education?

**DOI:** 10.1186/s12909-024-05564-2

**Published:** 2024-05-28

**Authors:** Amin Nakhostin-Ansari, Susan C. Mirabal, Thiago Bosco Mendes, Yuxing Emily Ma, Carolina Saldanha Neves Horta Lima, Kavita Chapla, Stasia Reynolds, Hannah Oswalt, Scott M. Wright, Sean Tackett

**Affiliations:** 1 Sports Medicine Research Center, Neuroscience Institute, Tehran University of Medical Sciences, Tehran, Iran; 2 Johns Hopkins University School of Medicine, Baltimore, MD USA; 3 University of Pittsburgh Medical Center, Pittsburgh, PA USA; 4 School of Medicine of University of São Paulo, São Paulo, Brazil; 5 Perelman School of Medicine, University of Pennsylvania, Philadelphia, USA; 6 Johns Hopkins Bayview Medical Center, Baltimore, MD USA; 7 Edward Via College of Osteopathic Medicine, Spartanburg, SC USA

**Keywords:** Education, Medical, Information dissemination, Journal article

## Abstract

**Background:**

The dissemination of published scholarship is intended to bring new evidence and ideas to a wide audience. However, the increasing number of articles makes it challenging to determine where to focus one’s attention. This study describes factors that may influence decisions to read and recommend a medical education article.

**Methods:**

Authors analyzed data collected from March 2021 through September 2022 during a monthly process to identify “Must Read” articles in medical education. An international team of health sciences educators, learners, and researchers voted on titles and abstracts to advance articles to full text review. Full texts were rated using five criteria: relevance, methodology, readability, originality, and whether it addressed a critical issue in medical education. At an end-of-month meeting, 3–4 articles were chosen by consensus as “Must Read” articles. Analyses were used to explore the associations of article characteristics and ratings with Must Read selection.

**Results:**

Over a period of 19 months, 7487 articles from 856 journals were screened, 207 (2.8%) full texts were evaluated, and 62 (0.8%) were chosen as Must Reads. During screening, 3976 articles (53.1%) received no votes. BMC Medical Education had the largest number of articles at screening (*n* = 1181, 15.8%). Academic Medicine had the largest number as Must Reads (*n* = 22, 35.5%). In logistic regressions adjusting for the effect of individual reviewers, all rating criteria were independently associated with selection as a Must Read (*p* < 0.05), with methodology (OR 1.44 (95%CI = 1.23–1.69) and relevance (OR 1.43 (95%CI = 1.20–1.70)) having the highest odds ratios.

**Conclusions:**

Over half of the published medical education articles did not appeal to a diverse group of potential readers; this represents a missed opportunity to make an impact and potentially wasted effort. Our findings suggest opportunities to enhance value in the production and dissemination of medical education scholarship.

**Supplementary Information:**

The online version contains supplementary material available at 10.1186/s12909-024-05564-2.

## Introduction

Publishing articles in peer-reviewed journals is intended to make rigorous scholarship available to a large audience [1, 2]. However, across fields, over 25% of published articles can go uncited [3–5] with estimates of 15% of articles in top-tier medical education journals going without any citations [6]. Even when articles are cited by other studies, they may not capture the attention of the intended audience or practitioners. The failure to translate the evidence from medical education’s primary literature into educational practice may indicate that many are unable to keep up with its innovations and discoveries [7, 8]. 

One reason that so much research receives little attention is the sheer volume of publications; article numbers have increased fivefold in the last 20 years [6]. Clinician-educators, in particular, face significant time constraints as they try to balance teaching responsibilities with clinical demands, administrative roles, and the need to pursue their own scholarly endeavors [9]. In clinical medicine, there are a variety of resources that curate and summarize the most important emerging evidence [10, 11], but there are no analogous services that regularly and systematically cull medical education scholarship.

If an article is not seen or read, then its discoveries, innovations, and ideas cannot be applied to practice and scholarship. While article citation patterns are routinely measured and studied, little is known about the reading patterns among those involved in medical education. While some journals offer data on the number of downloads and views for published articles, those measures are specific to the journal and do not provide insight into the reasons people decide to read the articles [12]. To our knowledge, there have been no systematic efforts to understand what may influence someone to read a medical education article or recommend it to others. Therefore, this study analyzed data collected as part of a monthly process undertaken to select Must Read articles in medical education. In this process, reviewers make a rapid judgment on whether to read an article, rate selected full texts, then choose 3–4 Must Read articles, which are recommended on a dedicated website [13] for individuals who are interested in medical education.

## Materials and methods

### Design

This study was a retrospective analysis of the data collected during the Must Read process from March 2021 to September 2022. While Must Read cycles are conducted each month, we focused on this 19-month period, because different rating scales for full-text review were in place before and after this time frame. This study was considered not human subjects research by a Johns Hopkins Medicine IRB on April 20, 2023(IRB00384814).

### Review process

#### Setting and process development

The review process was developed during 2019 and 2020 by appraising the peer-reviewed literature and consulting with experienced medical educators to consider how best to identify Must Read articles. We conducted six rounds of pilot cycles during the initial phase to refine the review process. After multiple iterations, we arrived at the final method to identify medical education Must Reads. This involved a systematic search, screening titles and abstracts, full text review, and a meeting to achieve consensus. The process was also guided by the wisdom of crowds model, where everyone’s opinions were independent and had the same value [14]. We limited the number of Must Reads to 3–4 each month based on feedback from pilot rounds, the effort required to create succinct article summaries, and technical considerations for the Must Reads website.

#### Reviewing team

The reviewing team consisted of health sciences educators, medical trainees, learners, and educational scholars from the U.S., Brazil, and Iran. Participation was voluntary, and the number of reviewers per cycle varied based on reviewer availability. Two individuals participated every month in order to provide consistency in the final selection of Must Reads across months; however screening and full text review were done independently and not influenced by any other reviewers.

#### Identifying articles

Each month, we searched PubMed using a search strategy developed in collaboration with an informationist and refined during our pilot cycles to retrieve medical education articles. In addition to searching for medical education articles in general, the search strategy specifically retrieved all articles published in the eight general medical education journals with the highest impact factor from Clarivate’s Journal Citation Reports (JCR) in the category of “Education, Scientific Disciplines [15]”: Academic Medicine, Advances in Health Sciences Education, BMC Medical Education, Journal of Surgical Education, Medical Education, Medical Teacher, Perspectives on Medical Education, and Teaching and Learning in Medicine. Perspectives on Medical Education was included in the search starting in February 2022 after it received a JCR impact factor. Throughout the study period, we also included the Journal of Graduate Medical Education, which does not have a JCR impact factor, but was felt to be important based on feedback during process development. We used PubMed’s filter to retrieve only articles with abstracts, and the search retrieved articles published two months prior to the search date to account for variations in journal indexing rates into PubMed. A sample of our search query for August 2022 is outlined in supplementary file 1.

#### Screening articles

Each cycle, all reviewers independently screened all titles and abstracts and voted yes or no using Covidence systematic review software (Veritas Health Innovation, Melbourne, Australia). The reviewers were instructed to vote quickly based on their interest in reading the article. All reviewer votes were tallied and those with the most votes were selected for full text review.

#### Full text review

During our development process, we recognized that 8–12 full text reviews achieved the desired compromise for expeditious and thorough review with the need to identify at least three articles to highlight as Must Reads. Each cycle, all reviewers independently rated each full text based on five criteria: relevance to a broad audience, methodological rigor, readability, originality, and whether it addressed a critical issue in medical education. Reviewers could rate each criterion as 1 (“not worth the time”), 2 (“good enough to read”), and 3 (“absolutely a Must Read”). Mean scores across all reviewers for each criterion and the total score were calculated each month prior to a 1-hour, online, end-of-month meeting where reviewers discussed their opinions about the articles and chose 3–4 Must Reads.

### Variables in the must read database

#### Core variables

Each month, for each article, we recorded the full citation information (including title, abstract, and author information), each reviewer’s votes during screening, and their ratings of full-text articles for each of the five criteria.

#### Additional variables

We retrieved the 2021 Scopus cite score for each journal from which an article was selected for full text review. The cite score is the average number of citations in the prior three years for each document in a journal according to the Scopus database and is updated annually [16, 17]. We used the cite score in our study as it is freely available and comparable to the JCR impact factor in evaluating citation rates [17]. In addition, for each full text we reviewed, we manually extracted the first author’s h-index and the country of their institutional appointment from Scopus in February 2023. We also recorded whether all authors were from the same country or two or more countries based on their affiliations.

### Statistical analysis

At the screening stage, we calculated the percentage of votes for each article as the number of positive votes divided by the number of reviewers for that month. We used Pearson’s correlation test to assess the relationship between the percentage of votes and word counts for article titles and abstracts.

When comparing full text variables associated with selection as a Must Read, we used the Mann-Whitney test to compare continuous and ordinal variables. Fisher’s exact or Chi-square tests were used as appropriate to compare categorical variables between groups. We used Pearson’s correlation to test the association between the percentage of Must Read articles from the nine medical education journals and their corresponding cite scores.

We performed a series of unadjusted binary logistic regressions that used each full text review criterion rating as the independent variable and selection as a Must Read article as the dependent variable. We also performed a multivariate regression using a mixed effects model that included all predictor variables as fixed effects and reviewers as random effects. For this model, we evaluated for collinearity between the criteria ratings using the variance inflation factor (VIF) and Pearson’s correlation test. A VIF of higher than 10 and a correlation coefficient of higher than 0.7 are considered indicators of the presence of collinearity, and their thresholds were not exceeded [18]. For ease of interpretation, we report unadjusted and adjusted odds ratios to correspond to a 0.1 increase in a criterion rating along its 1–3 scale.

All analyses were performed using R version 4.2.1 (2021-11-01). *P* < 0.05 was considered as statistically significant.

## Results

A total of 17 reviewers participated in reviewing and screening articles over the 19-month study period. The median number of reviewers for each cycle was 6 (IQR 5–8, min = 5, max = 9). The median number of cycles for each reviewer was 6 (IQR 2–9, min = 1, max = 19).

### Screening stage

From March 2021 to September 2022, 7487 articles from 856 journals were screened. There were 3976 articles (53.1%) that did not receive a positive vote from any reviewer, 3177 (42.4%) that received votes from at least one but fewer than half of the reviewers, and 334 (4.5%) that received votes from a majority of the reviewers. Four articles (0.05%) received votes from all reviewers. The median percentage of articles with no votes each month was 53.3% (IQR 49.7–57.4%, min = 40.3%, max = 66.5%). Reviewers who completed at least the median number of cycles (six cycles or more) voted to include a median of 14.2% of articles in the full-text review (IQR 13.0-19.3%, min = 7.4%, max = 20.6%).

The median title length was 14 words (IQR 11–17, min = 1, max = 42), and the median abstract length was 259 words (IQR 208–305, min = 7, max = 924). There were minimal correlations between the percentage of votes received during screening and shorter titles (*r*= -0.11, *p* < 0.001) and longer abstracts (*r* = 0.04, *p* < 0.001).

**Fig. 1:**
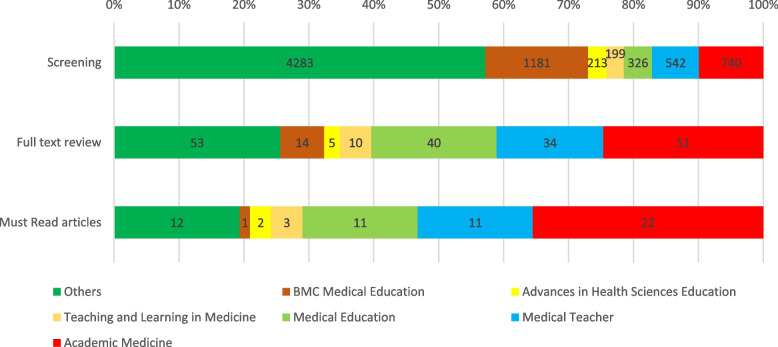
Numbers of articles from journals at each stage of the Must Read process The “Others” group encompasses articles from journals not specifically listed in the Figure. The number of journals in the “Others” group in the screening stage, full text review stage, and among Must Reads was 850, 38, and 12, respectively

The percentages of each journal’s articles under review at each stage of the Must Read process are presented in Fig. 1. BMC Medical Education had the greatest number of screened articles (*n* = 1181, 15.8%), followed by Academic Medicine (*n* = 740, 9.9%) and Journal of Surgical Education (*n* = 706, 9.4%). The journals with the greatest percentage of articles with at least one vote were Perspectives on Medical Education (*n* = 41/47, 87.2%), Medical Education (*n* = 283/326, 86.8%), and Teaching and Learning in Medicine (154/199, 77.4%).

### Full text review stage

The full texts of 207 (2.8%) articles were evaluated, and 62 (0.8%) were chosen as Must Reads. The percentage of reviewers’ votes during the screening stage did not differ between the articles chosen as Must Reads (median 66.7% of reviewers [IQR 62–80%, min = 50%, max = 100%]) and those not selected (median 66.7% of reviewers [IQR 60–75%, min = 42.9%, max = 100%]) (*p* = 0.18).

The characteristics of articles included in the full-text review are shown in Table 1. Most first authors were located in the US (*n* = 113, 54.6%), followed by Canada (*n* = 27, 13%), the UK (*n* = 21, 10.1%), and the Netherlands (*n* = 13, 6.3%). Comparing Must Read articles to other full texts, there was a significantly greater number of authors (median 6 (IQR 3–7) vs. median 4 (IQR 3–6)) (*p* = 0.04). Comparisons for title length, abstract length, number of references, first author’s h-index, and percentage of authors from two or more countries showed no statistically significant associations (*p* > 0.05).

**Table 1:** Characteristics of 207 articles included in the full-text review phase of the process where reviewers rated articles to determine if they would be designated as a Must Read

Article Variable		Must Read (*n* = 62)	Not a Must Read (*n* = 145)	*P*-value
Title word count, median (IQR)		13 (10-16)	12 (11-16)	0.821
Abstract word count, median (IQR)		280 (226–306)	274 (210–304)	0.447
Number of references, median (IQR)		49 (35–63)	43 (30–64)	0.366
First author’s h-index, median (IQR)		5 (2-12)	5 (2-11)	0.622
Number of authors, median (IQR)		6 (3-7)	4 (3-6)	0.037
Authors from ≥ 2 countries, n (%)		8 (12.9%)	38 (26.2%)	0.054
Country of the first author, n (%)	USA	34 (54.8%)	79 (54.5%)	0.463
	Canada	10 (16.1%)	17 (11.7%)	
	UK	7 (11.3%)	14 (9.7%)	
	Netherlands	5 (8.1%)	8 (5.5%)	
	Other	6 (9.7%)	27 (18.6%)	

*Abbreviations*: *IQR *interquartile range, *UK *United Kingdom, *USA *United States of America

Academic Medicine had the highest percentage of Must Read articles (*n* = 22, 35.5%). There was a positive correlation between the nine medical education journals’ cite scores and their percentage of Must Reads (*r* = 0.709, *p* = 0.03).

Must Read articles had significantly higher ratings across all criteria used for assessing articles in the full-text review stage in both the unadjusted and adjusted regressions (*p* < 0.05, Table 2). In the multivariate model that adjusted for reviewer effects, statistically significant independent associations with selection as a Must Read article were found for all criteria (Table 2).

**Table 2:** Full-text review ratings of selected and non-selected Must Read articles

	Ratings^a^ of each criterion, median (IQR)		OR^b^ (95% CI)	
Criteria	Must Read (*n* = 62)	Not a Must Read (*n* = 145)	Unadjusted	Adjusted
**Methodology**	2.00 (1.81–2.30)	1.80 (1.50- 2.00)	1.21 (1.11–1.33)	1.44 (1.23–1.69)
**Relevance**	2.29 (2.15–2.40)	1.89 (1.67–2.17)	1.47 (1.31–1.68)	1.43 (1.20–1.70)
**Readability**	2.23 (2.00-2.40)	2.00 (1.80–2.20)	1.24 (1.12–1.38)	1.37 (1.16–1.64)
**Originality**	2.07 (1.83–2.27)	1.83 (1.67-2.00)	1.21 (1.10–1.34)	1.22 (1.05–1.42)^c^
**Addresses a critical issue in medical education**	2.17 (2.00-2.33)	1.83 (1.67-2.00)	1.34 (1.20–1.51)	1.17 (1.00-1.36)^d^

*Abbreviations*: *CI *confidence interval, *IQR *interquartile range, *OR *odds ratio

^a^Ratings were from 1–3

^b^Odds ratios to correspond to a 0.1 increase in mean rating for each criterion. Adjusted odds ratios were from mixed-effects models that included all criteria in the model and adjusted for reviewers as random effects

All *p* < 0.001 except for ^c^*p* = 0.008 and ^d^*p* = 0.049

## Discussion

In this study, we analyzed a unique database to explore variables that may make medical education articles appealing to readers. Notably, many published articles did not interest any of the reviewers. Methodology and relevance to a broad audience were found to be the variables that were most strongly associated with an article being selected as a Must Read in medical education.

Despite including a diverse pool of reviewers, all of whom had significant curiosity and interest in medical education, approximately half of the articles retrieved in our search each month did not receive any votes in the screening stage. A study of top medical education journals reported that approximately 15% of articles published between 2000 and 2019 went uncited [6]. Studies from outside medical education have found that citation rates correlate with article downloads and reads from websites and reference managers [19]. Such metrics, however, fail to capture or quantify the articles that are being overlooked by potential readers. The monthly process in our study, which began with a search, followed by a rapid review of titles and abstracts, and ended in reading full texts, likely simulated a common approach by which both casual readers and educational scholars navigate the vast body of literature to find the articles that may inform their practices. Our methods are unique in that we were able to determine not only what was to be selected for reading, but also what was not.

The relatively high proportion of published medical education articles that were not judged to be interesting along with evidence that many articles go uncited may suggest that there is waste in the medical education scholarly enterprise. The factors responsible for the increasing quantity of published scholarship include promotion incentives, elevated status in academia afforded to those who publish more, and the greater array of outlets to publish with the advent of electronic-only journals [20, 21]. There are also prevalent factors that may lower the quality of medical education publications, such as limited funding and few opportunities for faculty development related to research [22–24]. The investment of time and resources devoted to publishing in medical education may need to be reconsidered; shifting incentives to support other educational activities, such as teaching and curricular enhancements, may promote greater value across the medical education system.

We found that journal practices may impact one’s ability to find an article they would like to read. For example, BMC Medical Education had the highest number of articles in the screening stage, but only one selected as a Must Read. BMC Medical Education is an open-access and online-only journal that makes its published articles widely available [25]. Academic Medicine had the greatest proportion of Must Read articles but rejects more than 90% of submitted articles [26], and much of its content may not be available without a journal subscription. Journals and editorial teams must make trade-offs when seeking to serve their readers and society. There may be a tension between creating a platform that is inclusive of diverse voices and perspectives while also excluding publications that do not adhere to the highest academic standards. Journals collectively appear to be struggling in meeting these goals, as evidence suggests an ongoing need to ensure greater representation among global voices [27, 28], while the volume of articles remains a major barrier for medical educators to stay up-to-date [29, 30]. Academic publishing, in general, is drawing scrutiny as profit motives have the potential to supersede scholarly values [31, 32]. As a community, we may need to look to improve the balance between ensuring equitable opportunity for all scholars without contributing to the unsustainable rise in article quantity.

Methodological rigor in medical education scholarship is discussed frequently; this includes debates about the value of different types of research [33], and the development and use of instruments designed to rate the quality of medical education studies [34–36]. Nearly 10 years ago, Sullivan and colleagues pointed out that the users of educational scholarship are rarely asked what they value [37]. There has been little additional work since, but the existing evidence suggests that readers may find the relevance and accessibility of an article to be more important than methodological rigor when seeking to apply findings to their own educational practices [29, 30]. In our study, we found significant heterogeneity in reviewers’ interests; few articles received votes from a majority of reviewers, and only a small fraction of all articles received votes from all reviewers. At the full text stage, nearly all criteria were deemed to be important to an article’s selection as a Must Read and the decision to recommend it to a broad audience. Our results emphasize the need to view quality in medical education scholarship through a multidimensional lens in order to enhance a publication’s reach and accessibility.

Several limitations of this study should be considered. First, while the search strategy was developed with an informationist and refined during piloting, we may not have retrieved all medical education articles. Our decision to include only articles with abstracts might also have excluded some noteworthy articles. Second, during the development process, we decided only 3–4 Must Read articles would be highlighted each cycle, a number we felt appropriate when recommending articles for busy educators. However, it was not always easy for our team to decide which articles were Must Reads, and many were worth reading. Third, while each review considered only one-month’s worth of publications, final selection of Must Reads was not completely independent of previous months; in selecting the Must Read articles, the team was mindful of articles or content areas that were recently shared in an effort to keep the growing Must Read collection diverse. Lastly, our findings reflect the opinions and priorities of the Must Reads reviewer team, and reviewers were not the same for each month. While we had representation across learners, educators, and researchers from three countries, and two reviewers participated every month to improve consistency across months, another group of individuals with different disciplines, experiences in medical education, and cultural backgrounds might have arrived at different selections. Particularly, we did not have a surgical or procedural specialist on our reviewing team, which may have limited interest in surgically-focused articles.

## Conclusions

Our study suggests that many medical education articles may not be capturing the attention or be appealing to those interested in medical education. This may serve as a call to action to enhance value in the production and dissemination of medical education scholarship.

### Supplementary Information

Supplementary Material 1.

## Data Availability

The data that support the findings of this study are available from the corresponding author, ANA, upon reasonable request.

## Abbreviations

IRB: Institutional Review Board
IQR: Interquartile range
JCR: Journal Citation Reports
VIF: Variance Inflation Factor
