# Supplementary material for: What makes an article a must read in medical education?
Source: BMC Med Educ. 2024 May 28;24:582. doi: 10.1186/s12909-024-05564-2 (PMC11134941; doi:10.1186/s12909-024-05564-2)
Supplement: Supplementary file 1 — Supplementary Material 1. [file 12909_2024_5564_MOESM1_ESM.docx]

**Sample of a search query for August 2022**

((("Education, Medical"[Majr] OR "medical education"[ti]) OR ("Education, Professional"[Majr:NoExp] OR "health professional education"[ti])) OR (((((((("Acad Med"[Journal]) OR "Med Educ"[Journal]) OR "Adv Health Sci Educ Theory Pract"[Journal]) OR "Med Teach"[Journal]) OR "Teach Learn Med"[Journal]) OR "J Surg Educ"[Journal]) OR "BMC Med Educ"[Journal]) OR "J Grad Med Educ"[Journal] OR "Perspectives on medical education"[Journal])) AND ("2022/6/01"[Date - Publication] : "2022/6/30"[Date - Publication]))
